# Modulatory mechanism of the paraventricular thalamus (PVT) under general anesthesia

**DOI:** 10.3389/fphar.2026.1732125

**Published:** 2026-01-14

**Authors:** Jia Li, Yiyong Wei, Donghang Zhang

**Affiliations:** 1 Department of Anesthesiology, Xi’an Honghui Hospital, Xi’an Jiaotong University, Xi’an, China; 2 Department of Anesthesiology, Longgang District Maternity and Child Healthcare Hospital of Shenzhen City (Longgang Maternity and Child Institute of Shantou University Medical College), Shenzhen, China; 3 Department of Anesthesiology, West China Hospital, Sichuan University, Chengdu, China

**Keywords:** circuits, general anesthesia, general anesthetics, paraventricular thalamus, PVT

## Abstract

The paraventricular thalamus (PVT) is a critical brain region involved in controlling sleep-wakefulness. Many neural nuclei and circuits regulate consciousness under both sleep-wakefulness and general anesthesia, suggesting that a common neural mechanism contributes to these two conditions. Recently, accumulating evidence has revealed that the activities of the PVT are associated with the actions of both volatile and intravenous general anesthetics. However, there is divergence regarding neuronal types, circuits, or different anesthesia periods. Herein, we reviewed the current literature and summarized the role of PVT in general anesthesia, which provides a better understanding of the modulatory mechanism of PVT on the actions of general anesthetics.

## Introduction

**FIGURE 1:**
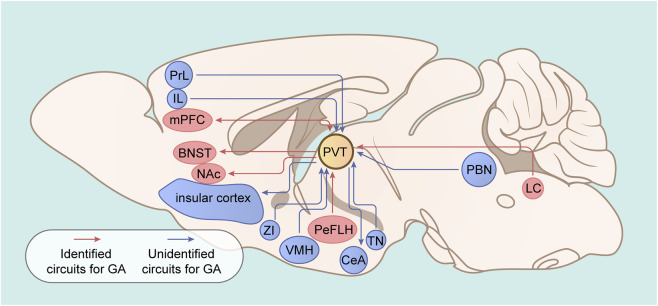
Identified or putative circuits involving the PVT under general anesthesia. The red arrow indicates identified PVT projections under general anesthesia; the blue arrow indicates putative PVT projections under general anesthesia. The LC-PVT is involved in the emergence but not the induction of isoflurane; the PeFLH-PVT is involved in the emergence of isoflurane and desflurane and the induction of desflurane but not of isoflurane; the PVT-mPFC is involved in the emergence and induction of sevoflurane; the PVT-BNST is involved in the emergence and induction of sevoflurane; and the PVT-NAc is involved in the emergence and induction of sevoflurane. The role of other circuits of the PVT was not determined under general anesthesia. PVT: paraventricular thalamus; NAc: nucleus accumbens; LC: locus coeruleus; PeFLH: perifornical area of the hypothalamus; BNST: bed nucleus of the stria terminalis; mPFC: medial prefrontal cortex; ZI: zona incerta; CeA: central amygdala; PBN: parabrachial nucleus; PrL: prelimbic cortex; IL: infralimbic cortex; VMH: ventromedial hypothalamus; TN: tuberal nucleus.

The paraventricular thalamus (PVT) is a key midline node of the dorsal thalamus involved in many behaviors, such as fear (Zhao et al., 2025), reward and motivation (Zorab et al., 2025; Minère et al., 2025), drug addiction (Zhu et al., 2023), feeding (Ye et al., 2023), homeostatic behavior (Ye et al., 2023; Penzo and Gao, 2021), and sleep-wakefulness (Ren et al., 2018). Clinical evidence has shown that injuries in the paramedian region of the thalamus (the homologous area of the rodent PVT) disrupt consciousness ranging from drowsiness to coma in patients (Hermann et al., 2008; Schmahmann, 2003; Luigetti et al., 2011). In rodents, inhibiting PVT activity suppressed consciousness, whereas increasing PVT activity facilitated wakefulness (Ren et al., 2018). These observations indicate that the PVT is a critical brain nucleus involved in controlling sleep-wakefulness. Interestingly, natural sleep and general anesthesia share the common characteristic of reversible unconsciousness, and many studies indicate that they are modulated via common neural mechanisms (Bao et al., 2023; Jiang-Xie et al., 2019; Yang et al., 2022). Recently, an increasing number of studies have investigated the role of PVT in general anesthesia. Their findings suggest that PVT is closely related to consciousness under volatile isoflurane (Bu et al., 2022; Ao et al., 2021), sevoflurane (Li et al., 2022; Wu et al., 2024), desflurane (Zhao et al., 2021), and intravenous propofol (Wang et al., 2023). Although the current evidence reaches the consensus that increased PVT activity accelerates emergence from general anesthesia, there is disagreement regarding the anesthesia induction process. Additionally, the involved neuronal populations and circuits also diverge. In 2024, Gao et al. (2024) reviewed recent advances in the neural mechanism of general anesthesia-induced unconsciousness. While this paper included descriptions of the PVT’s role in general anesthesia, it provided only a partial summary of optogenetic and chemogenetic findings and omitted several key studies (Ao et al., 2021; Wu et al., 2024; Zhao et al., 2021; Zhao et al., 2024) concerning the PVT’s involvement in general anesthesia. Therefore, it remains meaningful to conduct a focused review on the PVT’s specific role in general anesthesia. Here, we systematically searched databases (PubMed, Web of Science, Embase, the Cochrane Library, and Google Scholar) from inception to 15 August 2025, using the keywords “PVT,” “paraventricular thalamus,” “general anesthetics,” and “general anesthesia.” The objective was to specially summarize the modulatory effects of PVT under general anesthesia, independent of the experimental techniques employed (Table 1). We also illustrated the common brain circuits involved in PVT whose contributions are not currently known to occur during the regulation of general anesthesia (Figure 1). This study complies with the TITAN Guidelines 2025 for AI reporting (Agha et al., 2025).

**TABLE 1:** Study characteristics.

Study	Animals	Sex	Age (weeks)	Anesthetics	Main techniques	Circuits	Behaviroal outcomes	Effects on induction period	Effects on emergence period
Ren et al. (2018)	Mice	male^a^	6–8	Isoflurane	c-Fos staining; virus tracing; fiber photometry; multichannel Electrophysiological recordings; EEG-EMG; optogenetic and chemogenetic manipulation; behavioral tests	PVT^Glu^-NAc and LH^Hcrt^-PVT^Glu^ regulate wakefulness; no tests in isoflurane anesthesia	Emergence Time	Not available	Activation of PVT^Glu^ accelerates emergence
Ao et al. (2021)	Mice	Male	8–12	Isoflurane	c-Fos staining; optogenetic and chemogenetic manipulation; EEG; behavioral tests	LC^Th^-PVT	Induction and emergence Time	No change	Activation of the LC^Th^-PVT accelerates emergence
Zhao et al. (2024)	Rats	Male	​	Desflurane and isoflurane	c-Fos staining; optogenetic and chemogenetic manipulation; EEG; behavioral tests	PeFLH^Hcrt^-PVT	Induction and emergence Time	Activation of PeFLH^Hcrt^-PVT prolongs induction of desflurane, but not isoflurane	Activation of PeFLH^Hcrt^-PVT accelerates emergence from desflurane and isoflurane anesthesia
Li et al. (2022)	Mice	Male and female	8–10	Sevoflurane	c-Fos staining; virus tracing; optogenetic and chemogenetic manipulation; EEG Behavioral tests	PVT^Glu^-BNST	Induction and emergence Time; EC_50_ for LORR and RORR	Activation of PVT^Glu^ or PVT^Glu^-BNST prolongs the induction	Activation of PVT^Glu^ or PVT^Glu^-BNST accelerates the emergence
Bu et al. (2022)	Mice	Male	Adult	Isoflurane	c-Fos staining; fiber photometry; EEG; chemogenetic techniques; behavioral tests	​	Induction and emergence Time	No change	Activation of PVT^Glu^ accelerates emergence
Wang et al. (2023)	Mice	Male	6–16	Propofol	Fiber photometry; EEG-EMG; optogenetic and chemogenetic manipulation; behavioral tests	​	Induction and emergence Time; ED_50_ of LORR	Activation of PVT^Cr^ prolongs induction	Activation of PVT^Cr^ accelerates emergence
Duan et al. (2023)	Mice	Male	6–16	Isoflurane	c-Fos staining; fiber photometry; EEG; chemogenetic techniques; behavioral tests	​	Induction and emergence Time	Esketamine accelerates isoflurane induction, which can be abolished by suppression of PVT^Glu^	Esketamine accelerates emergence from isoflurane anesthesia, which can be abolished by suppression of PVT^Glu^
Wu et al. (2024)	Mice	Male and female	8	Sevoflurane	c-Fos staining; virus tracing; *in vivo* multichannel Recordings; EEG; optogenetic and chemogenetic manipulation; local field potentials; behavioral tests	PVT^Glu^-NAc	Induction and emergence Time; sevoflurane concentration at LORR and RORR	Activation of PVT^Glu^ prolongs induction	Activation of PVT^Glu^ accelerates emergence
Zhao et al. (2021)	Mice	Male and female	8	Sevoflurane	c-Fos staining; virus tracing; *in vivo* multichannel Recordings; EEG; single-cell RNA sequencing; optogenetic and chemogenetic manipulation; local field potentials; behavioral tests	PVT^Astro^-PVT^Chat^-PFC	Induction and emergence Time; sevoflurane concentration at LORR and RORR	Activation of PVT^Chat^ prolongs induction; activation of PVT^Astro^ did not affect induction	Activation of PVT^Chat^ accelerates emergence; activation of PVT^Astro^ accelerates emergence

^a^ Female mice were also used for virus tracing and *in vitro* electrophysiological experiments.

EEG: electroencephalography; EMG: electromyography; PVT^Glu^: paraventricular thalamus glutamatergic neurons; PVT^Chat^: paraventricular thalamus chat-expressing neurons; PVT^Astro^: paraventricular thalamus astrocytes; NAc: nucleus accumbens; LH^Hcrt^: hypocretin neurons in the lateral hypothalamus; LC^Th^: locus coeruleus tyrosine-hydroxylase neurons; PeFLH^Hcrt^: perifornical area of the hypothalamus orexinergic neurons; EC_50_: the concentration at which 50% of the mice lose or recover their righting reflex; BNST: bed nucleus of the stria terminalis; LORR: loss of righting reflex; RORR: recovery of righting reflex; PVT^Cr^: PVT, calretinin neurons.

### Role of PVT in volatile anesthesia

#### Isoflurane

In 2018, Ren et al. (2018) first reported that PVT activity was related to consciousness under isoflurane general anesthesia. The authors detected higher levels of c-Fos expression in the PVT than in the other regions of the paramedian thalamus after extended wakefulness in mice. *In vivo* fiber photometry and multichannel electrophysiological recordings revealed increased activity in the glutamatergic neurons of the PVT (PVT^Glu^) during wakefulness. The results from electroencephalography (EEG) recordings and spectral analysis revealed that chemogenetic suppression of the activity of the PVT^Glu^ reduced wakefulness. Conversely, optogenetic activation of the PVT^Glu^ induced wakefulness from sleep. They further investigated whether manipulation of the activities of the PVT^Glu^ influences wakefulness from an unconscious state induced by isoflurane. Optogenetic activation of the PVT^Glu^ induced a transition from anesthesia to emergence in EEG signals and significantly shortened the emergence time from isoflurane anesthesia. Additionally, they searched for the upstream and downstream pathways through which the PVT modulates wakefulness. Hypocretin neurons in the lateral hypothalamus to the PVT^Glu^ and PVT^Glu^ to the nucleus accumbens projections are indicated as effector pathways for wakefulness control. Although they reported that the PVT^Glu^ also sends projections to the medial prefrontal cortex (mPFC) and insular cortex, these projections were not found to be involved in the control of wakefulness. However, they did not investigate the role of these projections in isoflurane general anesthesia. Moreover, it is not clear whether the activities of the PVT^Glu^ are also associated with the isoflurane induction period. Notably, only male mice were used for the behavioral experiments, and whether sex differences exist in the wakefulness control of the PVT needs to be further determined.

Bu et al. (2022) reported similar findings concerning the role of the PVT in isoflurane anesthesia. The authors observed lower expression of c-Fos in the PVT during the isoflurane anesthesia period than during recovery or wakefulness in male mice. Using *in vivo* fiber photometry, they reported that the activity of the PVT^Glu^ was suppressed under isoflurane anesthesia but increased during emergence from anesthesia. Chemogenetic activation of the PVT^Glu^ accelerated the emergence of isoflurane anesthesia. Conversely, chemogenetic inhibition of the PVT^Glu^ delayed the emergence of isoflurane anesthesia. Interestingly, the induction time was not affected, although the cortical EEG was changed during isoflurane induction. However, measurement of the median effective concentration for loss of the righting reflex is needed to confirm the role of PVT in isoflurane induction. This study did not explore the upstream or downstream region of the PVT^Glu^ that modulates isoflurane anesthesia.

One study (Ao et al., 2021) identified the upstream pathway through which the PVT regulates isoflurane anesthesia in male mice. Ao and colleagues reported that tyrosine hydroxylase-positive neurons in the locus coeruleus (LC^Th^) were activated during emergence from isoflurane anesthesia. Chemogenetic activation of LC^Th^ induced cortical arousal in EEG signals and promoted emergence from isoflurane anesthesia, with concurrent c-Fos upregulation in the PVT. Optogenetic activation of the LC^Th^-PVT shortened emergence time and induced EEG arousal, whereas chemogenetic inhibition produced the opposite results. Therefore, these findings reveal a new pathway that regulates emergence from isoflurane anesthesia. However, whether the LC^Th^-PVT regulates the induction process of isoflurane anesthesia has not been determined. Moreover, the subtypes of neurons in the PVT that receive LC^Th^ inputs under isoflurane anesthesia have not been determined. Additionally, whether other neuronal subtypes of the LC are involved in isoflurane anesthesia remains unclear.

#### Sevoflurane

Li et al. (2022) investigated the role of the PVT and its involved circuits in sevoflurane general anesthesia using both male and female mice. They reported that c-fos expression in the PVT was inhibited by sevoflurane exposure. Chemogenetic inhibition of the PVT^Glu^ shortened the sevoflurane induction time and prolonged the emergence time while suppressing cortical EEG arousal during the sevoflurane maintenance period. However, optogenetic activation of the PVT^Glu^ produced the opposite results. The authors further explored the pathways downstream of the PVT that mediate the effects of sevoflurane anesthesia. Using virus tracing techniques, they reported that the PVT^Glu^ projected to both glutamatergic and gamma-aminobutyric acid (GABAergic) neurons in the bed nucleus of the stria terminalis (BNST). And this pathway was confirmed to modulate the effects of sevoflurane anesthesia. However, it remains unclear whether sex differences exist in the regulation of this projection under sevoflurane anesthesia. Moreover, they did not investigate the functions of PVT-BNST glutamatergic neurons or PVT-BNST GABAergic neurons separately under sevoflurane anesthesia.

Similarly, Wu and colleagues (Wu et al., 2024) demonstrated that PVT glutamatergic neuronal activities were suppressed during both sevoflurane induction and maintenance phases using c-fos staining and *in vivo* multiple-channel recordings. A gradual recovery of these neuronal activities was observed during the emergence period from anesthesia. They further identified the sodium leak channel (NALCN) as a key molecular target that mediates the effects of sevoflurane on the activities of PVT glutamatergic neurons. Additionally, PVT glutamatergic neurons to the nucleus accumbens constitute one circuit that regulates the actions of general anesthesia (Wu et al., 2024). Consistently, this circuit also modulates consciousness levels during the sleep-wakefulness cycle (Ren et al., 2018). However, the neuronal subtypes of the nucleus accumbens that receive PVT inputs have not been determined. Moreover, the direct effects of sevoflurane on the NALCN of PVT glutamatergic neurons were not tested via patch-clamp electrophysiological techniques. Zhao et al. (2024) used single-cell RNA sequencing and reported that Chat-expressing neurons are the key subpopulation of PVT glutamatergic neurons (PVT^Chat^) that mediate the actions of sevoflurane. They reported that Kir4.1 in astrocytes regulated the activities of PVT^Chat^, which contributed to the emergence-promoting effects of PVT via projection to the prefrontal cortex (Zhao et al., 2024). Interestingly, the modulatory effects of PVT^Chat^ on basic physiological functions remain largely unknown and need to be determined in future studies. Collectively, these two (Wu et al., 2024; Zhao et al., 2024) studies revealed the underlying molecular mechanism by which PVT regulates sevoflurane anesthesia.

#### Desflurane

The orexinergic system is suggested to be involved in the regulation of emergence from general anesthesia induced by several types of anesthetics, such as isoflurane (Kelz et al., 2008; Li et al., 2019; Wang et al., 2021), sevoflurane (Kelz et al., 2008), and propofol (Zhang et al., 2012). Zhao et al. (2021) explored the role of orexinergic neurons in the PVT in male rats under desflurane anesthesia. Using c-Fos staining, they reported that the activity of orexinergic neurons in the PeFLH (PeFLH^Hcrt^) was inhibited by desflurane anesthesia. The activity of orexinergic neurons in downstream nuclei, including the PVT, basal forebrain (BF), dorsal raphe nucleus (DRN), and ventral tegmental area (VTA) was also suppressed. Chemogenetic activation of PeFLH^Hcrt^-PVT prolonged the induction time and reduced the emergence time from desflurane anesthesia, whereas chemogenetic inhibition produced the opposite results. They also investigated whether the PeFLH^Hcrt^-PVT projection regulates isoflurane anesthesia. Similarly, chemogenetic activation of this projection decreased the emergence time from isoflurane anesthesia, whereas chemogenetic inhibition increased the emergence time. Notably, manipulation of the PeFLH^Hcrt^-PVT projection did not influence isoflurane induction. Finally, they reported that OX2Rs are the receptors in the PVT that receive inputs from PeFLH^Hcrt^ to regulate the effects of desflurane anesthesia. However, researchers have not determined whether other downstream nuclei of the PeFLH, such as the BF, DRN, and VTA, are involved in the regulation of desflurane anesthesia. The neuronal populations in the PVT that received the inputs of the PeFLH orexinergic neurons were not identified.

### Role of PVT in intravenous general anesthesia

#### Propofol

In addition to volatile anesthetics, the PVT also regulates the actions of intravenous anesthetics according to recent evidence. Wang et al. (2023) investigated the contribution of PVT to intravenous propofol anesthesia in male mice. Using *in vivo* fiber photometry, they observed decreased activity of PVT^Glu^ following propofol induction in mice. This activity rapidly recovered during emergence, suggesting that PVT^Glu^ was likely involved in the mechanism of action of propofol. They further tested the causal role of calretinin-expressing neurons, a major subset of PVT glutamatergic neurons (PVT^Cr^), under propofol anesthesia. Chemogenetic or optogenetic inhibition of the PVT^Cr^ shortened the propofol induction time and prolonged the recovery time, whereas chemogenetic activation of the PVT^Cr^ produced the opposite results. During the maintenance period of propofol anesthesia, optogenetic activation of the PVT^Cr^ induced cortical activation according to EEG and increased behavioral arousal. It will be interesting to determine the role of other neuronal subtypes of PVT in propofol anesthesia. Furthermore, the upstream and downstream targets of the PVT^Cr^ that regulate propofol anesthesia need to be further investigated.

#### Esketamine

Duan et al. (2023) investigated the role of the PVT in the regulation of emergence from isoflurane anesthesia by esketamine. C-fos staining and *in vivo* fiber photometry revealed that the activity of the PVT^Glu^ was elevated by a low dose of esketamine during isoflurane anesthesia. Interestingly, esketamine promoted both the induction of and recovery from isoflurane anesthesia, which could be abolished by chemogenetic suppression of the PVT^Glu^. These findings suggest that the rapid recovery from general anesthesia induced by esketamine is mediated by activation of the PVT^Glu^. This finding may be intriguing because esketamine is well known as an antagonist of N-methyl-D-aspartate receptors. The inhibition of GABAergic interneurons by esketamine may explain this contradictory mechanism. Future studies need to investigate the molecular or circuitry mechanism by which esketamine activates the PVT^Glu^. Importantly, the complicated mechanism by which esketamine also accelerates isoflurane induction was not well explored or explained in this study. It remains unclear whether esketamine affects the induction and emergence process of intravenous anesthesia or other inhalational anesthesia, such as sevoflurane. Together with findings from previous studies, these findings suggest that the PVT is likely a common target for intravenous and inhalational general anesthesia. It will be interesting to determine whether the PVT is also involved in the actions of other intravenous anesthetics, such as etomidate, ketamine, and dexmedetomidine.

## Discussion

Current evidence demonstrates that the activity of the PVT^Glu^ plays a pivotal role in regulating emergence from both volatile and intravenous general anesthesia, suggesting that the PVT may serve as a common target of general anesthetics. However, the contribution of PVT to the induction process of general anesthesia remains controversial. Most studies indicate no association between PVT activity and isoflurane anesthesia induction (Bu et al., 2022; Ao et al., 2021; Zhao et al., 2021) because manipulation of PVT activity does not influence induction time. Like these findings in the PVT, several brain regions or circuits that selectively affect the emergence period from isoflurane but not the induction process have been identified; these regions include the parabrachial nucleus–lateral hypothalamus or the parabrachial nucleus–BF circuit (Lu et al., 2023), serotonergic neurons in the DRN (Yang et al., 2019; Li et al., 2021), dopaminergic neurons in the VTA (Li et al., 2019), the BF (Zhang et al., 2016), orexinergic neurons in the perifornical hypothalamus (Kelz et al., 2008), and the olfactory tubercle (Yang et al., 2021). Therefore, other regions that affect both isoflurane induction and emergence may contribute more than the PVT to isoflurane induction, such as the ventrolateral preoptic nucleus (Moore et al., 2012), supraoptic nucleus (Jiang-Xie et al., 2019), and ventral periaqueductal gray (Liu et al., 2020). Interestingly, one study revealed that GABAergic transmission in the pontine reticular formation influenced the induction of propofol or isoflurane but not emergence in rats (Vanini et al., 2014). Nevertheless, studies have revealed that the PVT is involved in both the induction and emergence of sevoflurane ^16 17^, desflurane (Zhao et al., 2021), and propofol (Wang et al., 2023) anesthesia. These results suggest that the molecular targets within the PVT or its upstream and downstream circuits involved in the induction of anesthesia by isoflurane differ from those involved in the process of other anesthetics. These findings indicate that induction is not a simple reverse process of recovery for isoflurane, at least in terms of the mechanism related to PVT. Hysteresis/neural inertia is suggested to be the underlying reason (Friedman et al., 2010). Hysteresis/neural inertia demonstrates an intrinsic tendency of the central nervous system to resist transitions from unconscious to conscious states, which cannot be solely explained by pharmacokinetics (Joiner et al., 2013). We speculated that the PVT might be an important area contributing to hysteresis/neural inertia under isoflurane-induced general anesthesia. Notably, poor experimental methods might influence the accuracy of the data. For example, high doses of general anesthetics might induce a rapid loss of the righting reflex, which makes it difficult to detect a notable difference in the induction time. Furthermore, a lack of temperature monitoring or thermal insulation measures under general anesthesia can cause hypothermia and influence the assessment. Therefore, well-designed studies are still needed to confirm the role of PVT in isoflurane induction.

The specific molecular mechanism by which general anesthetics commonly suppress the activities of PVT^Glu^ remains largely unknown because existing studies on the role of PVT in volatile and intravenous anesthesia remain limited. It is suggested that the ion channels and receptors expressed in the PVT might serve as important targets of general anesthetics. Recent evidence has shown that NALCN of glutamatergic neurons and Kir4.1 channels in astrocytes are key molecular targets that mediate the effects of sevoflurane on the activity of the PVT (Wu et al., 2024). More future studies are needed to clarify the molecular mechanisms. In addition, although the LC-PVT (Ao et al., 2021), PVT-BNST (Li et al., 2022), PVT-nucleus accumbens (Wu et al., 2024), and PVT-PFC (Zhao et al., 2024) circuits have been shown to regulate the effects of volatile anesthesia, the upstream and downstream pathways through which the PVT controls consciousness levels under general anesthesia are largely unknown. However, whether the abovementioned circuits regulate the general anesthesia induced by propofol is unclear. Future studies need to determine whether the classical circuits involved in the PVT, such as the mPFC-PVT (Lucantonio et al., 2021; Yamamuro et al., 2020), PVT-central amygdala (Ma et al., 2021; Keyes et al., 2020), and zona incerta-PVT (Ye et al., 2023; Zhang and van den Pol, 2017) (Figure 1), are associated with the actions of general anesthetics. Notably, other influences might exist between various PVT circuits, such as the diverse subpopulations and subregions in the PVT and its upstream and downstream targets (Kirouac, 2025), the various ion channels or receptors expressed in neurons or glial cells of the PVT and its involved circuits (Wu et al., 2024; Zhao et al., 2024; Mulkey et al., 2022), and potential sex differences (Zhang et al., 2022; Braithwaite et al., 2023). Furthermore, the interactions between various PVT circuits and the tertiary projection loop remain largely unknown and need to be elucidated in future studies. For example, sleep-promoting projections might inhibit the activities of PVT-involved circuits and weaken their effects on consciousness levels under general anesthesia (Ren et al., 2024). Given PVT circuits’ involvement in diverse physiological functions, assessing whether PVT manipulation induces abnormal phenotypes (e.g., respiratory or cardiovascular changes) is crucial. Such phenotypic alterations could potentially influence the actions of general anesthetics.

Despite accumulating animal evidence of the involvement of the PVT in emergence from general anesthesia, data concerning the contribution of the PVT to general anesthesia in humans are limited. Notably, emerging clinical evidence highlights the therapeutic potential of another thalamic region-the subthalamic nucleus (STN). Specifically, deep brain stimulation (DBS) of the STN has been established as an effective treatment for patients with Parkinson’s disease experiencing disabling motor response fluctuations (Hol et al., 2021; Roldán et al., 2025; de Dos Reis Paula et al., 2024). Furthermore, DBS can be safely performed under general anesthesia with reduced procedure duration compared to awake states (Gadot et al., 2023). However, no clinical studies have yet investigated the effects of DBS on consciousness levels during general anesthesia. This gap presents an intriguing opportunity to explore whether DBS targeting the PVT in comatose patients could enhance consciousness under anesthesia, given that clinical studies have reported that injuries in the thalamic paramedian region (the homologous area of the rodent PVT) lead to decreased consciousness levels or coma in patients (Hermann et al., 2008; Schmahmann, 2003; Luigetti et al., 2011) (Bassetti et al., 1996; Montagna et al., 2002). These findings underscore the translational potential of PVT-related mechanisms from rodent models to clinical applications. For example, monitoring PVT activity may provide a more sensitive indicator of general anesthesia depth than bispectral index value. More importantly, direct stimulation of the human PVT might facilitate emergence from delayed general anesthesia or from coma. However, the inhibition of PVT activity might reduce the consumption of general anesthetics and opioids, which will decrease the related complications and is safer for debilitated patients. Notably, a recent study (Bian et al., 2021) showed that noninvasive ultrasound stimulation of the ventral tegmental area facilitated mouse emergence from isoflurane anesthesia, suggesting the possibility of noninvasive manipulation of the PVT in clinical patients.

## Limitations

Several limitations exist in our work. First, existing studies on the role of PVT in volatile and intravenous anesthesia remain limited, more future studies are needed to clarify the molecular mechanisms. Second, sex differences might be notable in the regulation of PVT in general anesthesia, evidenced by that males are more sensitive to general anesthetics than females (Zhang et al., 2022; Braithwaite et al., 2023). Brain regions are suggested to be implicated in the sex differences in response to anesthetics. For instance, the estrogen receptor alpha of the medial preoptic area contributes to the differentially sensitive to sevoflurane between male and female mice (Zhang et al., 2022). Current study had yet investigated whether sex differences exist in the regulation of PVT in general anesthesia. Finally, despite progress in elucidating the anesthetic mechanisms of PVT in animal models, its clinical translation faces multiple challenges. There are significant structural and functional differences between the human and mouse PVT, making research findings from animal models difficult to directly apply to humans. Moreover, there is a lack of precise technical methods to target specific neuronal populations in the human PVT, and techniques for real-time monitoring of PVT neuronal activity in clinic remain underdeveloped. Additionally, direct intervention in the PVT may lead to unpredictable neurological side effects. Of note, studies on consciousness regulation will undergo rigorous ethical scrutiny. These factors constitute the predominantly practical barriers to clinical translation.

## Conclusion and perspectives

On the basis of the literature, we conclude that the PVT serves as a key emergence-promoting brain region under both volatile and intravenous general anesthesia. However, current evidence regarding the pivotal role of PVT in general anesthesia primarily come from rodent studies, and clinical data about the structural and functional aspects of the human PVT are urgently needed to facilitate the translation. The molecular mechanisms by which PVT contributes to the regulation of general anesthesia remain largely unknown, though recent studies have identified NALCN in PVT glutamatergic neurons and Kir4.1 in PVT astrocytes as potential targets for general anesthetics. Future studies will aim to identify more specific targets, such as ion channels, neurotransmitters as well as the receptors that modulate the actions of general anesthetics.
